# Circadian Patterns of Patients with Type 2 Diabetes and Obstructive Sleep Apnea

**DOI:** 10.3390/jcm10020244

**Published:** 2021-01-11

**Authors:** Trinitat Cambras, Odile Romero, Antoni Díez-Noguera, Albert Lecube, Gabriel Sampol

**Affiliations:** 1 Department of Biochemistry and Physiology, Faculty of Pharmacy and Food Sciences, Universitat de Barcelonan, 08028 Barcelona, Spain; cambras@ub.edu (T.C.); adieznoguera@ub.edu (A.D.-N.); 2 Multidisciplinary Sleep Unit, Neurophysiology & Respiratory Departments, University Hospital Vall d’Hebron, 08035 Barcelona, Spain; gsampol@vhebron.net; 3 CIBER of Respiratory Diseases (CIBERES), Institute of Health Carlos III (ISCIII), 28029 Madrid, Spain; 4 Department of Endocrinology and Nutrition, University Hospital Arnau de Vilanova, 25198 Lleida, Spain; alecube@gmail.com; 5 Obesity, Diabetes and Metabolism Research Group (ODIM), Biomedical Research Institute of Lleida, (IRBLleida), Universitat de Lleida, 25198 Lleida, Spain; 6 CIBER of Diabetes and Associated Metabolic Diseases (CIBERDEM), Institute of Health Carlos III (ISCIII), 28029 Madrid, Spain

**Keywords:** glycated hemoglobin, circadian, apnea-hypoapnea index

## Abstract

Sleep apnea, a condition that modifies sleep and circadian rhythms, is highly prevalent in patients with diabetes. However, it is not known if there is an association between sleep apnea, circadian alterations and glycemic regulation in this type of patient. Here, a polysomnographic study was carried out on 21 women and 25 men (mean age = 64.3 ± 1.46 years) with diagnoses of type 2 diabetes to detect the presence of sleep apnea. Moreover, patients wore an actigraph and a temperature sensor on the wrist for one week, to study the manifestation of the circadian rhythms. The correlations of circadian and polysomnographic variables with the severity of apnea, measured by the apnea-hypopnea index, and with glycemic dysregulation, measured by the percentage of glycated hemoglobin, were analyzed. The mean apnea-hypoapnea index of all the participants was 39.6 ± 4.3. Apnea-hypoapnea index correlated with % N1, negatively with % N3, and also the stability of the active circadian rhythm. However, no significant correlation was found between the apnea-hypopnea index and wrist temperature rhythm and glycated hemoglobin. Glycated hemoglobin levels were negatively associated with the percentage of variance explained by the wrist temperature circadian rhythm (calculated via 24 and 12 h rhythms). This association was independent of body mass index and was strongest in patients with severe apnea. In conclusion, patients with diabetes showed altered circadian rhythms associated with a poor glycemic control and this association could partially be related to the coexistence of sleep apnea.

## 1. Introduction

Diabetes is a highly prevalent disease throughout the world associated with high morbidity and mortality [1]. To avoid the microcardiovascular complications associated with diabetes, an adequate control of the levels of glycemia is necessary. Glycosylated hemoglobin (HbA1c) levels could be used as an objective measure of glycemic regulation which, according to the American Diabetes Society values, are recommended to be under 7% [2].

In recent years sleep apnea-hypopnea syndrome (SAHS) has been described to be highly prevalent in patients with type 2 diabetes (T2D), from a lowest estimate of 58% to a highest estimate of 86% [3]. Moreover, epidemiological studies have shown the association between diabetes and short sleep duration or poor-quality sleep [4,5,6]. Sleep disturbances are strongly related to circadian alterations, which in turn have also been related to abnormal glucose metabolism and increased diabetic risk [3].

The circadian system is responsible for the coordination of most daily processes and it is formed by the central clock located in the hypothalamic suprachiasmatic nuclei, which receives light information directly from the retina, and a network of peripheral clocks present in practically all tissues [7]. The central clock sends its signals to the peripheral clocks through the autonomic nervous system and hormonal signals, among others. Moreover, peripheral clocks are also regulated by food and are closely related to metabolic cycles [8]. Circadian misalignment, specially observed in shift workers [9], has been related to metabolic alterations and to the presence of diabetes, probably due to alterations in glucose metabolism and levels of circulating hormones [10,11]. Additionally, obesity, a common feature among patients with diabetes, also alters circadian rhythms [12,13], which may in turn contribute to the alteration of the circadian manifestation in patients with diabetes. Thus, since circadian impairment has been considered a risk for metabolic syndrome, we here wanted to study to which extent glycemic regulation is related with circadian alterations in patients with type 2 diabetes and, also if glycemic regulation could be impaired by sleep apnea in this type of patient.

We here hypothesized that sleep apnea could worsen glycemic control in diabetic patients, and that this glycemic dysregulation would be related with the impairment of the circadian rhythmicity. With this purpose, we have studied sleep structure, activity and skin temperature rhythms, as well as biochemical variables in a group of patients with T2D, with and without sleep apnea.

## 2. Materials and Methods

### 2.1. Participants

Forty-eight consecutive patients with diagnoses of type 2 diabetes submitted to the sleep unit (Sleep Unit, Vall d’Hebron University Hospital, Barcelona, Spain) for suspected sleep apnea were prospectively enrolled. Type 2 diabetes was diagnosed according to the American Diabetes Association criteria [2] (American Diabetes Association, 2018). Antidiabetic therapy included oral and subcutaneous agents. Exclusion criteria were: shift work, pregnancy, psychiatric disease, clinical instability, current or previous SAHS treatment, treatment with sedatives, melatonin, antidepressants or other medication that could interfere with sleep. The study was approved by the Ethical Committee on Human Research of the participating centers and patients provided written informed consent prior to participation.

### 2.2. General Procedure

All individuals attended the hospital for a blood analysis after a night fasting. This day participants were asked to complete a sleep diary and to wear, during one week, an ambulatory device, an actigraph (MotionWatch8, CamNtech, UK) on the wrist of the non-dominant arm and a temperature sensor (Thermochrom IButton^®^ device, DS1921H, Maxim integrated, USA) fixed on the same wrist with elastic tape. Subjects were asked to continue with their usual daily activities during the seven day period of actigraphy and temperature monitoring. Activity data were recorded at 1 min intervals and temperature data every 5 min. After one week, subjects went back to the hospital for a polysomnography study and returning the devices.

### 2.3. Physical Examination

In the initial visit, height, weight and waist circumference measured at the halfway level between the lower rib and iliac crest were determined, and body mass index (BMI) was calculated. Systolic and diastolic blood pressure (BP) were measured with a digital sphygmomanometer after 5 min in a sitting position. The lower of two consecutive measurements was recorded.

### 2.4. Polysomnography

All participants underwent a full overnight polysomnography (PSG) at the Sleep Unit of our institution with a Compumedics E Series system, (Abbotsford, Victoria, Australia). The recorded parameters included electroencephalography (EEG), electro-occulography (EOG), electromyography (EMG), electrocardiography (ECG), respiratory effort, oronasal airflow using a thermistor and nasal cannula, finger pulse oximetry, snoring sounds, and body position. Sleep scoring was performed according to the American Academy of Sleep Medicine (AASM) scoring criteria 2012.

Apnea was defined as a decrease of 90% in pre-event baseline airflow for at least 10 s detected by the oronasal thermal sensor. A differentiation between obstructive and central apneas was made according to respiratory effort channels (the presence or absence of thoracoabdominal movement). Hypopnea was defined as a ≥30% reduction in flow amplitude with respect to the baseline using a nasal cannula pressure sensor for duration of at least 10 s and associated with either a drop in basal oxygen saturation of at least 3%. The apnea-hypopnea index (AHI) was calculated as the number of apneas plus hypopneas per hour of sleep.

### 2.5. Blood Samples

Blood samples were analyzed in the normal routine of the analytical laboratory of the hospital and, among other variables, glucose and glycosylated hemoglobin (HbA1c) were determined.

### 2.6. Circadian Variables

Data obtained by means of actigraphy and the temperature sensor were analyzed using “El-temps, v313”, an integrated package for chronobiological analysis (A. Díez-Noguera, University of Barcelona; http://www.el-temps.com). Data of each variable (activity and wrist temperature) were adjusted to a 24 h sinusoidal curve and the mean 24 h value (mean), the acrophase (time of the day when the variable has its maximum value after adjustment to a cosinusoidal curve) and the amplitude of the adjusted 24 h rhythm (A_cos) were calculated. Moreover, non-parametric circadian analysis was also performed as previously described [12,14,15,16,17]. In such a way, M10 and L5 intervals, which denoted the mean levels of activity during the 10 most active and the 5 least active hours, respectively, were calculated. In the case of wrist temperature, due to the inverse pattern of this variable compared with activity, we calculated M5 and L10, which denoted the mean temperature values of the 5 h with highest temperature and that of the 10 h with lowest temperature. In addition, the intradaily variability (IV) was studied as a measure of rhythm fragmentation, and the stability of the rhythm was measured by the percentage of variance explained by the 24 h rhythm (PV_wave) and by the daily stability of acrophases measured by Rayleigh test (R).

Additionally, to study the pattern of the 24 h profile, a spectral analysis was carried out with the mean waveform of each individual. Individual mean daily profile was obtained by averaging data of the seven days of the recording for each subject, and the power spectrum of the mean waveform was calculated with two harmonics. The power content of the first harmonic, P1, was an indicator of the importance of the 24 h rhythm; the power of the 12 h rhythm (second harmonic), P2, was used as an indicator of temperature post-noon peak or activity post-noon decrease. Moreover, the power content of the first two harmonics simultaneously, P1 + 2, was used for comparisons of daily profile as a whole, indicating the percentage of the variance explained by the circadian rhythm.

### 2.7. Statistical Analysis

Continuous variables were summarized as mean ± standard error and were compared using ANOVA (for data normally distributed) or Kruskal–Wallis test (not normally distributed); *p* values of less than 0.05 were considered statistically significant. Acrophases were compared by means of the Watson–Williams test for circular statistics. To address the relationship between the different variables, partial correlations were carried out controlling for confounding variables. Moreover stepwise multiple regression analyses were carried out to explore the effect of circadian variables on diabetes severity. In this case, HbA1c was considered the dependent variable and the independent variables included in the analysis were the circadian variables: A_cos, IV, PV_wave, R, M5 or M10, L10 or L5, P1, P2 and P1 + 2 calculated with activity and temperature data separately. Standardized beta values are presented. Statistical analysis was carried out using IBM SPSS statistics (IBM SPSS Statistics for Windows, Version 22.0. Armonk, NY: IBM Corp, USA).

## 3. Results

From the 48 patients with type 2 diabetes, HbA1c values were missed in two of them. Moreover, from another subject data temperature were not usable. From the total of 46 patients, AHI had a mean value of 39.6 ± 4.3. Five patients had an AHI < 5, five had an AHI between 6 and 15; ten had an AHI between 15 and 28 and twenty-six an AHI > 30.

### 3.1. Sleep Apnea Modifies Polysomnography and Circadian Activity Rhythm of Patients with Type 2 Diabetes

First of all, patients were classified taking into account the severity of the apnea into two groups: patients with none, mild or moderate apnea (AHI < 30; *n* = 20) and with severe apnea (AHI > 30; *n* = 26). In Table 1, summary statistics describing the characteristics of the participants stratified by the two groups are shown.

Clinical variables indicated that the group with severe apnea showed higher BMI and had greater alterations in the nocturnal oxygenation variables and sleep variables (%N1 and %N3). The circadian variables obtained from the activity rhythm (R, P1 and P1 + 2) also differ among groups. However, in the case of the temperature rhythm, none of the circadian variables differed among groups. Additionally, no differences in glycemia or HbA1c levels were found between the two groups of patients.

**Figure 1:**
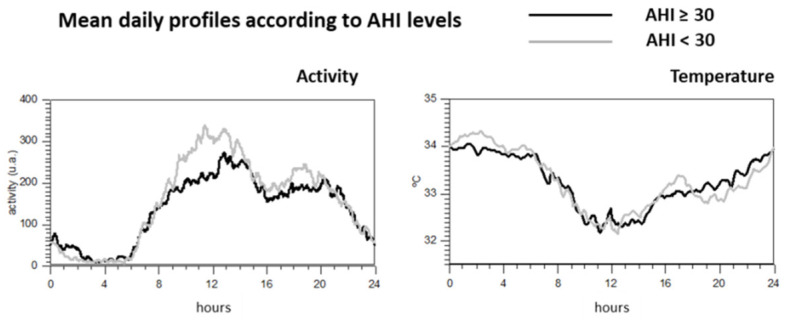
Mean daily profile of the activity and wrist temperature according to the levels of AHI.

Figure 1 illustrates the different circadian patterns between those patients according to the degree of apnea when activity or wrist temperature variables are studied. As observed, patients with higher AHI values showed different circadian activity profile, while no apparent differences in wrist temperature rhythm were found.

### 3.2. Glycemic Disregulation Associates with Circadian Rhythms of Patients with Type 2 Diabetes

Since the purpose of our study was to study the relationship between glycemic dysregulation and circadian manifestation in patients with diabetes, we carried out regression analysis considering HbA1c as the dependent variable and sleep and circadian variables as predictors. As BMI is a critical variable for T2D, apnea and circadian manifestation, we used BMI as a possible confounding variable.

First of all, it should be indicated that Pearson’s correlation revealed no statistically significant correlation between AHI and HbA1c (r = 0.03; *p* = 0.984), neither between AHI and glycemia (r = −0.181; *p* = 0.250).

We carried out partial correlations to test association between HbA1c with each of the sleep and circadian variables, controlling for BMI as a confounding variable. Results indicate that HbA1c failed to correlate with any of the sleep variables, but significantly correlated with some of the circadian variables (Table 2). In the case of activity rhythm, HbA1c was associated with R and P2. In the case of temperature rhythm, HbA1c was associated with P1 and P1 + 2 of the temperature rhythm. It is of interest to comment that when AHI was considered as a confounding variable, these associations practically were not modified. It is also interesting to note that these associations were maintained, even with higher significance, when only patients with AHI > 30 were considered, but not in the case of subjects with AHI < 30 (Table 2).

In the model, HbA1c was the dependent variable and the possible predictors were BMI, sex, A_cos, IV, PV, R, M10 (M5 for temperature rhythm), L5 (L10 for temperature rhythm), P1, P2 and P1 + 2. The final selection of the variables after automatic stepwise process showed that when the activity rhythm was considered, the predictors for HbA1c were R (*p* = 0.013) and the power of the second harmonic (12 h rhythm) of the spectrum (*p* = 0.018). In the case of the wrist temperature rhythm, HbA1c was negatively associated with the percentage of variance explained by a two-harmonic model, P1 + 2 (*p* = 0.001). These results were confirmed when multiple regression analysis with stepwise method based on the *p*-value of F was carried out (Supplementary Tables S1 and S2).

**Figure 2:**
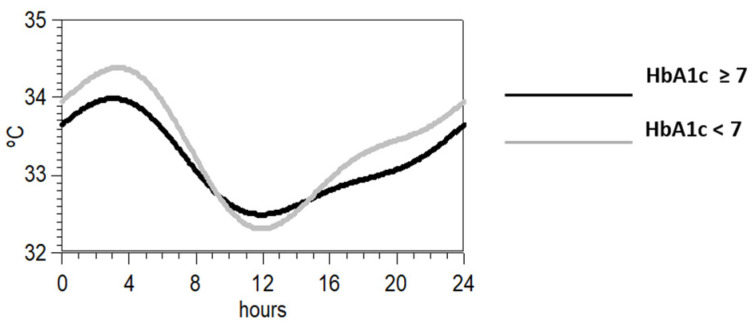
Synthesis of the mean waveform for wrist temperature according to a two-harmonic model of those individuals with HbA1c above (black) or below the mean (grey).

Since the most statistically significant difference between the groups with low and high levels of HbA1c was the variable P1 + 2 of the wrist temperature rhythm, we plotted in Figure 2 the rhythm profile of the temperature rhythm synthesized by means of a two- harmonic model. As it can be observed, those individuals with values lower than the mean showed more pronounced circadian rhythm.

**Figure 3:**
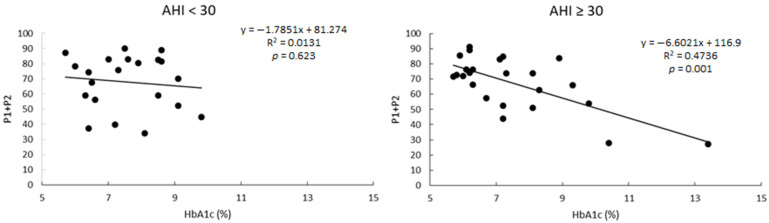
Linear correlations between the percentage of variance explained by a two-harmonic model (P1 + P2) and the levels of HbA1c in patients grouped as a function of the AHI levels.

It is of interest that correlations between P1 + P2 and HbA1c level were not significant in the group of patients with AHI < 30, but it was when only patients with severe apnea were considered (Figure 3).

## 4. Discussion

Population and clinical studies have found that poor sleep, a common cause of alterations in circadian rhythms, is frequent in patients with diabetes and has been associated with fluctuations in hormone levels and impairment of glucose metabolism [10,18,19,20]. However, these studies have not evaluated the role of sleep apnea, also a frequent comorbidity in patients with diabetes, as a factor associated with circadian rhythms’ disruption and its impact on HbA1c levels.

Our study demonstrates that the levels of HbA1c are related to an impairment of the stability of the circadian rhythmicity, and that this relationship is stronger in patients with severe sleep apnea. Moreover, it indicates that the evaluation of the circadian rhythmicity may depend on the studied variable (activity or wrist temperature) and that each variable provides different information, which can make one or another more suitable for the study of patients with diabetes or sleep apnea.

Our study of patients with T2D agrees with others in the sense that AHI was associated with sleep variables, as can be observed, since %N1 increased and %N3 stages decreased while AHI increased. This fits with the observation that AHI was correlated with less stability of the circadian rhythms, which is also compatible with poor sleep quality. However, we did not find that severe apnea modified sleep efficiency or sleep onset latency or total sleep time, which could be due to the fact that all individuals suffered from T2D. Actually, total sleep time had a mean of 5 h 48 min from a total of 8 h in bed.

On the other hand, HbA1c levels did not correlate with sleep variables, neither with AHI. However, it is of interest that HbA1c correlated with the stability of the circadian rhythms and with the percentage of variance explained by a two-harmonic model of wrist temperature, especially in the case of those individuals with severe sleep apnea. This suggests that although patients with T2D may suffer from sleep disorders, when sleep apnea and T2D coexist, the effect of T2D on sleep is masked by the effect of the apnea.

Nocturnal increments in glucose have been described in patients with sleep apnea [21], and high AHI or intermittent hypoxia indices have been related with poorer glycemic control [22,23]. On the other hand, some studies propose that T2D might contribute to the development of sleep breathing disorders [24].

It is of interest to note that alterations in circadian rhythms in patients with T2D and sleep apnea were associated with higher HbA1c values and that this association was independent of BMI, a known cause of rhythms alteration [12] and also of poor diabetes control.

HbA1c has been associated with severity of obstructive sleep apnea-hypopnea syndrome in nondiabetic men [25] and a correlation between SAHS severity and glycemic control has been previously reported [22,23]. Moreover, autonomic dysfunctions in sleep apnea related to the presence of T2D and SAHS has been already demonstrated [26,27]. However, in our study there is lack of correlation between the AHI and desaturation indices with HbA1c values. This could be due to the fact that all participants in this study had T2D and no controls were included.

It is of interest to notice that when we studied differences of circadian rhythms related to HbA1c, these differences were more significant when WT was used, suggesting that the shape of the daily pattern of the skin temperature is more unstable in patients with high HbA1c, but not in those patients with low AHI.

Although HbA1c levels are associated with circadian alterations, whether T2D exerts a negative impact on circadian rhythms is difficult to assess in our cross-sectional study. Perhaps the sympathetic activation present in T2D in response to intermittent hypoxia may increase the risk of the circadian abnormalities in patients with SAHS [28] and especially using wrist temperature. Moreover, we should also consider that other mechanisms than the presence of sleep apnea or T2D potentially involved in circadian rhythm alterations have been involved in glucose homeostasis, such as poor-quality sleep, meal timing, obesity and light exposition [29].

Although circadian rhythm alteration secondary to the presence of T2D has been studied [30], studies of human circadian rhythms have shown that under the standard 24 h daily cycle, the circadian pattern is not purely sinusoidal, but is modelled by a 12 h component (second harmonic), which has been related to the interaction between the homeostatic and circadian sleep regulatory components [31]. The presence of this 12 h rhythm is noticeable by a post-noon peak in wrist temperature rhythm or by a post-noon dip in activity. Alteration of this 12 h rhythm in humans has been found in several pathological situations [12,17,32]. It is worth noting that patients with higher levels of HbA1c display abnormalities in the adjustment to a model that includes the 12 h component.

We should take into account that alterations in the wrist temperature do not necessarily reflect alterations in the circadian pacemaker but in the regulation of the diameter of vascular vessels, which is regulated by the autonomous nervous system through hypothalamic signals from the central pacemaker [33]. There is evidence that patients with T2D and with SAHS may suffer more complications, including peripheral neuropathy, than those patients without SAHS [34]. This may be because SAHS is associated with activation of the sympathetic nervous system and of inflammatory processes as well as oxidative stress [28]. Perhaps the high levels of HbA1c would induce alterations in the endothelium, which could alter vasodilation/vasoconstriction capacity of the vessels. This would be in accordance with alterations in thermoregulation found in patients with diabetes [35,36,37]. Thus, SAHS might contribute to the development or increase of the severity of microvascular complications, which are associated with poor long-term glycemic control. Similarly, HbA1c has been related to increased arterial thickening and thus, poor glycemic control has been suggested to increase atherosclerosis risk [38]. Taking all this into account, the study of wrist temperature in poor glycemia-controlled patients might contribute to understanding the vascular alterations in these patients.

This study has certain limitations when interpreting our findings, starting with its cross-sectional nature, which prevent us finding causation. Moreover, we acknowledge about the relatively small number of individuals in the groups and the lack of healthy controls. Furthermore, there could be additional factors with potential impact on circadian rhythms, such as the chronotype of the individuals, which has not been considered and could be also involved in glucose metabolism [39]. Additionally, objective sleepiness was not studied and, given the known limitations of the ESS, its presence in our patients cannot be ruled out and could be involved in the lower activity detected in the severe apnea group. Nonetheless, our study has several strengths, such as the inclusion of participants with both T2D and SAHS, the simultaneous recording of sleep and actigraphic variables and considering BMI as a potential confounder.

In conclusion, we found out that alterations of circadian rhythms are associated with poorer glycemic control, especially in those individuals with severe apnea. Moreover, our study suggests that the impact of sleep apnea on diabetes control may be produced in part through the alteration of circadian rhythms. However, future research must determine if these alterations are secondary to circadian pacemaker activity or whether they are produced by microvascular complications of diabetes or by increased sympathetic activity influencing the regulation of peripheral vascular reactivity.

## Figures and Tables

**Table 1:** Demographic, clinical, polysomnographic and circadian variables of the participants (patients with type 2 diabetes) stratified in two groups according to the level of apnea-hypopnea index (AHI) (SEM: standard error). *p* indicates the significance obtained by Student’s t test. Significant differences are indicated in bold.

Variables	AHI < 30; *n* = 20		AHI > 30; *n* = 26		*p* Value
	Mean	SEM	Mean	SEM	
Clinical variables					
Age	66.15	2.00	62.92	2.05	0.276
Waist circumf. (cm)	104.36	2.43	115.43	2.30	**0.002**
BP_S	148.10	4.36	144.50	3.79	0.536
BP_D	79.90	2.38	85.92	1.61	**0.036**
BMI	28.54	0.60	33.84	0.84	**0.001**
Hours in bed (h)	8.00	0.29	8.01	0.25	0.982
TST	347.15	13.36	352.19	17.75	0.830
Sleep efficiency	70.31	3.12	71.62	3.57	0.791
Sleep onset latency	32.60	7.67	29.60	7.03	0.776
ESS	7.63	1.09	6.53	3.84	0.434
%N1	18.61	1.70	33.48	3.59	**0.001**
%N2	50.58	2.62	43.22	2.71	0.063
%N3	18.38	2.58	10.79	1.81	**0.021**
%REM	13.42	1.54	12.51	1.47	0.906
Arous./h	22.17	2.46	49.23	4.20	**0.000**
AHI	14.35	1.90	59.08	4.73	**0.000**
ODI3%	9.69	1.60	51.68	4.78	**0.000**
CT90%	3.24	1.09	9.97	1.61	**0.001**
SaO2 basal	95.55	0.34	95.65	0.22	0.796
PLMS	5.66	2.64	15.42	5.87	0.185
HbA1c (%)	7.59	0.26	7.44	0.35	0.736
Glycemia mg/dL	160.47	12.98	137.78	10.78	0.187
**ACTIVITY RHYTHM**					
mean	157.22	13.20	137.56	12.41	0.288
A_cos	137.89	13.14	109.09	11.01	0.098
IV	0.01	0.00	0.01	0.00	0.054
PV_Wave	46.45	1.85	39.88	2.51	0.052
R	0.95	0.01	0.88	0.02	**0.031**
M10	262.01	22.68	220.63	19.71	0.175
L5	11.76	1.01	13.93	2.06	0.394
P1	59.31	3.31	49.49	3.37	**0.047**
P2	13.12	1.89	14.13	1.88	0.711
P1 + 2	72.43	2.17	63.62	2.83	**0.018**
**WRIST TEMPERATURE RHYTHM**					
mean	33.50	0.22	33.46	0.17	0.949
A_cos	0.93	0.14	0.67	0.08	0.292
IV	0.10	0.02	0.12	0.02	0.668
PV_Wave	46.43	4.85	34.65	2.84	0.129
R	0.65	0.10	0.63	0.04	0.985
M5	34.61	0.17	34.22	0.18	0.540
L10	32.81	0.28	32.94	0.20	0.738
P1	56.91	5.36	47.97	4.78	0.272
P2	16.01	3.75	17.99	3.53	0.405
P1 + 2	72.93	3.95	65.96	3.49	0.467

Clinical variables: BP_S: systolic blood pressure; BP_D: diastolic blood pressure; BMI: body mass index; TST: total sleep time in minutes; ESS: Epworth Sleepiness Scale; %N1, %N2, %N3, %REM: percentage of TST for each sleep stage; arousal/h: number of arousal per hour; AHI: apnea-hypopnea index; ODI3: 3% oxygen desaturation index; T90: sleep time with SpO2 < 90%; SaO2: basal oxygen saturation; PLMS: periodic limb movements during sleep; HbA1c: glycosylated hemoglobin. Circadian variables: A_cos: amplitude; IV: intradaily variability; PV_Wave: interdaily stability; R: stability of acrophases; M10 (or M5): mean value of the 10 (or 5) consecutive hours with highest values. L5 (or L10): mean value of the 5 (or 10) consecutive hours with lowest values. P1: power content of the first harmonic (24 h); P2: power content of the second harmonic (12 h); P1 + 2: percentage of variance explained by a two-harmonic model.

**Table 2:** *p* Values obtained from Pearson correlation between HbA1c and circadian variables obtained with activity and wrist temperature (WT) data. Correlations were carried out considering BMI as a confounding variable. Significant differences are indicated in bold.

Correlation of HbA1c with:	Variable	Mean	A_cos	IV	PV	R	M5 or M10	L10 or L5	P1	P2	P1 + 2
All patients	activity	0.170	0.187	0.796	0.246	**0.025**	0.129	0.395	0.338	**0.017**	0.691
	WT	0.925	0.106	0.715	0.123	0.107	0.374	0.626	**0.049**	0.728	**0.003**
Patients with AHI < 30	activity	0.602	0.713	0.347	0.588	0.413	0.603	0.257	0.916	0.512	0.682
	WT	0.691	0.848	0.939	0.709	0.188	0.492	0.812	0.952	0.369	0.623
Patients with AHI ≥ 30	activity	0.204	0.227	0.949	0.174	**0.014**	0.160	0.525	0.432	**0.030**	0.666
	WT	0.847	**0.049**	0.695	0.064	**0.005**	0.558	0.433	**0.020**	0.918	**0.001**

## Data Availability

The data presented in this study are available on request from the corresponding author. The data are not publicly available due to the signed consent agreements around data sharing, which only allow access to the researchers of the study following the project purposes. Requestors wishing to access the data used in this study can make a request to O.R. The request will be subjected to approval and formal agreements regarding confidentiality and secure data storage being signed the data would be the provided.

## Supplementary Materials

The following are available online at https://www.mdpi.com/2077-0383/10/2/244/s1, Table S1: Multiple correlation analysis of HbA1c (glycosylated hemoglobin) as dependent variable with the circadian variables obtained by activity or wrist temperature data. Model 1: Multiple regression analysis. Model 2: Final model after stepwise process. Table S2: Multiple correlation analysis of HbA1c (glycosylated hemoglobin) as dependent variable with the circadian variables obtained by activity or wrist temperature data considering only those individuals with AHI > 30. Model 1: Multiple regression analysis. Model 2: Final model after stepwise process.
