# Supplementary material for: Circadian Patterns of Patients with Type 2 Diabetes and Obstructive Sleep Apnea
Source: J Clin Med. 2021 Jan 11;10(2):244. doi: 10.3390/jcm10020244 (PMC7826782; doi:10.3390/jcm10020244)
Supplement: Supplementary file 1 [file jcm-10-00244-s001.pdf]

Journal of
Clinical Medicine

Supplementary material

Table S1: Multiple correlation analysis of HbA1c (glycosylated hemoglobin) as dependent variable with the circadian variables obtained by activity or wrist temperature data. Model 1: Multiple regression analysis. Model 2. Final model after stepwise process.

Activity	Unstandardized coefficients		Standardized coefficients	t	Sig.
	B	SEM	Beta		
Model 1					
(Constant)	10.738	4.173		2.573	0.014
mean	−0.040	0.044	−1.607	−0.905	0.372
A_cos	−0.036	0.043	−1.377	−0.833	0.411
IV	61.565	86.168	0.128	0.714	0.480
PV_Wave	−0.015	0.031	−0.110	−0.475	0.637
R	−4.822	3.624	−0.332	−1.331	0.192
M5	0.047	0.045	3.179	1.051	0.301
L10	−0.012	0.040	−0.065	−0.292	0.772
P2	−0.036	0.040	−0.212	−0.899	0.375
P1+2	0.006	0.028	0.054	0.224	0.824
Model 2					
(Constant)	12.842	1.788		7.183	0.000
R	−5.014	1.934	−0.345	−2.592	**0.013**
P2	−0.055	0.022	−0.328	−2.463	**0.018**

Wrist temperature	Unstandardized coefficients		Standardized coefficients	t	Sig.
	B	SEM.	Beta		
Model 1					
(Constant)	14.501	12.225		1.186	0.244
mean	−4.794	4.667	−2.380	−1.027	0.312
A_cos	2.948	6.699	0.823	0.440	0.663
IV	−0.793	3.051	−0.043	−0.260	0.796
PV_Wave	−0.006	0.040	−0.059	−0.147	0.884
R	−0.925	1.129	−0.139	−0.819	0.418
M5	0.734	2.739	0.381	0.268	0.790
L10	3.992	5.439	2.382	0.734	0.468
P2	−0.008	0.029	−0.075	−0.268	0.791
P1+2	−0.058	0.024	−0.669	−2.411	**0.021**
Model 2					
(Constant)	10.284	0.822		12.506	0.000
P1+2	−0.041	0.012	−0.468	−3.470	**0.001**

Clinical variables: BP_S: systolic blood pressure; BP_D: diastolic blood pressure; TST: total sleep time; %N1, %N2, %N3, %REM: percentage of TST for each sleep stage; arousal/h: number of arousals per hour; ODI3: 3% oxygen desaturation index; T90: sleep time with SpO2 < 90%; SaO2: basal oxygen saturation; PLMS: periodic limb movements during sleep; **Circadian variables**: A_cos: amplitude; RA_np: non-parametric amplitude; IV: intradaily variability; PV_Wave: interdaily stability; R: stability of acrophases; M10 (or M5): mean value of the 10 (or 5) hours with maximum values; L5 (or L10): mean value of the 10 (or 5) hours with minimum values; P1: power content of the first harmonic (24h); P2: power content of the second harmonic (12h); P1+2: percentage of variance explained by a two-harmonic model.

Table S2: Multiple correlation analysis of HbA1c (glycosylated hemoglobin) as dependent variable with the circadian variables obtained by activity or wrist temperature data considering only those individuals with **AHI > 30.** Model 1: Multiple regression analysis. Model 2. Final model after stepwise process.

Activity	Unstandardized coefficients		Standardized coefficients	t	Sig.
	B	SEM	Beta		
Model 1					
(Constant)	9.522	4.256		2.237	0.040
mean	−0.097	0.089	−3.492	−1.093	0.291
A_cos	−0.052	0.068	−1.655	−0.769	0.453
IV	141.533	98.925	0.307	1.431	0.172
PV_Wave	−0.046	0.044	−0.335	−1.053	0.308
R	−6.034	4.903	−0.429	−1.231	0.236
M5	0.093	0.086	5.311	1.079	0.297
L10	0.012	0.049	0.072	0.242	0.812
P2	−0.028	0.062	−0.150	−0.449	0.660
P1+2	0.035	0.045	0.289	0.789	0.442
Model 2					
(Constant)	13.646	2.010		6.789	0.000
R	−9.671	2.493	−0.687	−3.878	**0.001**
P1	0.047	0.018	0.461	2.605	**0.016**

Wrist temperature	Unstandardized coefficients		Standardized coefficients	t	Sig.
	B	SEM.	Beta		
Model 1					
(Constant)	−0.309	14.879		−0.021	0.984
mean	−9.022	7.018	−4.472	−1.285	0.217
A_cos	10.003	10.162	2.441	0.984	0.340
IV	−1.454	3.834	−0.079	−0.379	0.709
PV_Wave	−0.019	0.058	−0.155	−0.325	0.749
R	−3.705	1.668	−0.456	−2.221	**0.041**
M5	−0.637	3.769	−0.329	−0.169	0.868
L10	10.092	8.337	5.860	1.210	0.244
P2	0.009	0.039	0.092	0.228	0.822
P1+2	−0.071	0.033	−0.716	−2.147	**0.047**
Model 2					
(Constant)	12.417	1.093		11.363	0.000
R	−2.735	1.315	−0.337	−2.080	**0.049**
P1+2	−0.049	0.016	−0.496	−3.064	**0.005**

Clinical variables: BP_S: systolic blood pressure; BP_D: diastolic blood pressure; TST: total sleep time; %N1, %N2, %N3, %REM: percentage of TST for each sleep stage; arousal/h: number of arousal per hour; ODI3: 3% oxygen desaturation index; T90: sleep time with SpO2 < 90%; SaO2: basal oxygen saturation; PLMS: periodic limb movements during sleep. **Circadian variables**: A_cos: amplitude; RA_np: non-parametric amplitude; IV: intradaily variability; PV_Wave: interdaily stability; R: stability of acrophases; M10 (or M5): mean value of the 10 (or 5) hours with maximum values; L5 (or L10): mean value of the 10 (or 5) hours with minimum values; P1: power content of the first harmonic (24h); P2: power content of the second harmonic (12h); P1+2: percentage of variance explained by a two-harmonic model.
